# Microscopy

**Published:** 1869-06-15

**Authors:** 


***Microscopy.***

The State Microscopical Society of Illinois is now well established. Its membership numbers many of the most prominent and respected Illinoisans, with a sprinkling of honorary, corresponding and auxiliary associates. The following are its officers:

President—Dr. W. W. Allport.

Vice Presidents—Hosmer A. Johnson, M.D., George F. Rumsey, James V. Z. Blaney, M.D., J. F. Beaty.

Treasurer—George M. Higginson.

Secretary—J. Hankey.

Secretary, Foreign Correspondence—Samuel A. Briggs.

Council—S. A. Briggs, Joseph T. Ryerson, N. S. Davis, M.D., J. Hankey, W. C. Hunt, M.D., W. E. Dogget, J. H. Hollister, M .D., J. F. Beaty, Walter Hay, M.D., R. Ludlam, M.D., Samuel J. Jones, M. D., George M. Higginson.

Curator—H. F. Monroe.

Librarian—John Robson.

Photomicrographer to the Society—John Carbut.

The Society now numbers fifty-six resident members, one associate member, and as

Corresponding Members—Lieut. Col. J. J. Woodward, war department, Washington; Christopher Johnson, M.D., Professor of Principles and Practice of Surgery, University of Maryland, Baltimore; Prof. H. L. Smith, Hobart College, Geneva, N. Y.; J. B. McQuillen, M.D., Philadelphia.

Honorary Members—W. S. Sullivant, Columbus, Ohio; W. H. Wamsley, Philadelphia, Pa.; Rev. R. H. Walker, late of Wadham College, Oxford, Enhland.

The objects of the Society are the promotion and extension of microscopical knowledge in the West, and more particularly in our own State, by associating those together engaged in such pursuits, whether for purely scientific purposes or for recreative or educational purposes.

The Society extends the right hand of fellowship to the geologist, the chemist, the mineralogist. the anatomist, and the botanist—all of whom find the microscope a useful companion and an indispensable aid in their interesting researches.

